# Rapidly involuting congenital haemangioma (RICH) associated with transient thrombocytopenia and coagulopathy^⋆^

**DOI:** 10.1016/j.abd.2022.06.014

**Published:** 2023-12-21

**Authors:** Ana María Palma, Tamara Gracia-Cazaña, Carmen Ruiz de la Cuesta-Martín, Yolanda Gilaberte

**Affiliations:** a Dermatology Service, Hospital Miguel Servet, Zaragoza, Spain; b IIS Aragon, Zaragoza University, Zaragoza, Spain; c Pediatric Service, Hospital Miguel Servet, Zaragoza, Spain

Dear Editor,

A full-term male infant was born by natural delivery, with a vascular tumor in the right thigh of 10 × 5 cm in diameter, with central ulceration and no adhered to deep planes, since birth (Fig. 1). He was transferred to the neonatal intensive care unit at 3 hours of life after detecting hypoprothrombinemia (24% prothrombin activity), prothrombin time: 36.8 seconds (range: 9-12), no signs of hemolytic anemia, normal bilirubin, and normal platelet count, he was treated with vitamin K and 2 infusions of fresh frozen plasma. He presented with moderate thrombocytopenia (60 × 109/L) on the fourth day of life, which remitted along with rapid involution of the tumor. At 2 weeks of life, the tumor has completely resolved leaving a residual subcutaneous atrophy.

**Figure 1:**
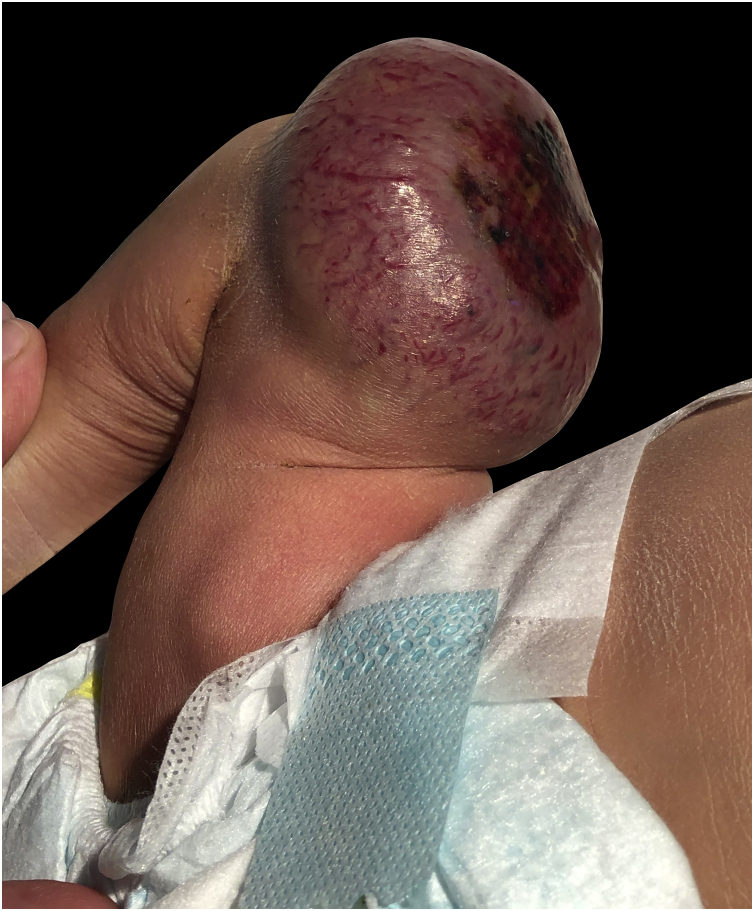
10 × 9 cm vascular tumor, image taken at birth, necrotic ulcerated plaque is observed on the surface of the tumor.

He was treated from birth with prednisone 2 mg/kg/day for 5 days with withdrawal after improvement of the tumor. Given the clinical picture of a congenital vascular tumor with rapid involution, the diagnosis of rapidly involuting congenital hemangioma (RICH) was made, with no need for a biopsy.

With the diagnosis of RICH-associated coagulopathy, the patient has been followed up for 8 months with a very important regression of the lesion (Fig. 2).

**Figure 2:**
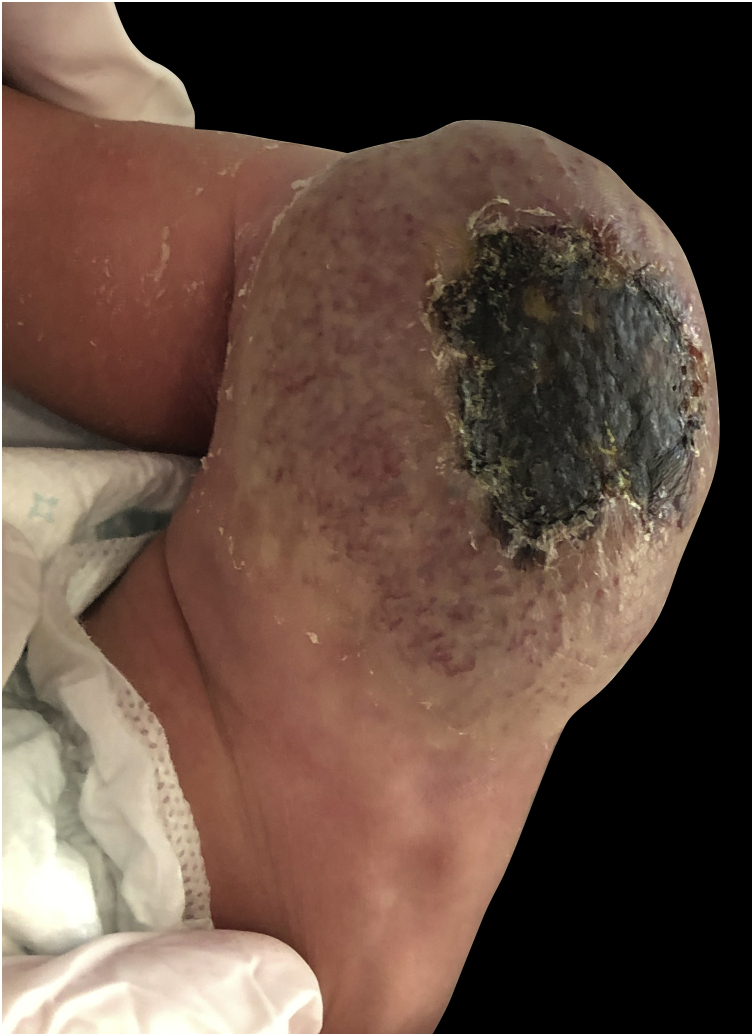
Progressive decrease in the volume of the tumor was seen 6 months of subsequent follow-up in consultation, disappearing ulceration with central necrosis and a persistent redundant skin plaque with residual surface telangiectasia.

## Discussion

Coagulopathy and thrombocytopenia are complications that may appear in some vascular tumors, especially when they reach a large volume, due to thromboembolic complications and potential hemodynamic repercussions. The main differential diagnosis that should be considered in this patient is Kasabach-Merritt Syndrome (KMS), a life-threatening thrombocytopenic coagulopathy associated with rare vascular tumors, such as Kaposiform hemangioendothelioma, and less frequently with tufted angioma but not with common infantile or congenital hemangiomas.^1^

Unlike the persistent coagulopathy seen with KMS,^1^ the thrombocytopenia which appears in RICH commonly does not disturb coagulation factors or is not as pronounced, and then normalizes in the first month of life.^2^

In the literature published as RICH and transient thrombocytopenia, there are only 11 cases described, including the case that we have reported, all of them are summarized in Table 1.^1^, ^3^, ^4^, ^5^

**Table 1:** Summary of all cases published to date of RICH-type hemangioma and associated thrombocytopenia.

	Sex	Glut-1	Size	Location	Platelet	Start of thrombo-cytopenia	Resolution of thrombocytopenia	Clinical signs of regression of hemagioma	Treatment
Baselga et al.^1^	Male	Negative	6 × 7 × 1.5 cm	Scalp	56 (×10^9^ L^–1^)	Day 5	14 days	6 months (resected)	Surgical removal
Baselga et al.^1^	Female	Negative	8 × 8 cm	Arm	62 (×10^9^ L^–1^)	Day 4	14 days	14 days	Oral PDN for 4 days and embolization
Baselga et al.^1^	Male	Negative	NA	Leg	5 (×10^9^ L^–1^)	Day 2	7 days	5 days	Oral PDN, 1 month; platelet transfusion day 2
Baselga et al.^1^	Male	NA	8 × 5 × 2.5 cm	Thorax	7 (×10^9^ L^–1^)	Day 1	30 days	NA	Embolization
Baselga et al.^1^	Male	NA	5.1 × 4.1 cm	Thigh	19 (×10^9^ L^–1^)	Day 2	11 days	11 days	Intravenous dexamethasone, oral PDN
Baselga et al.^1^	Female	NA	10 × 8 cm	Arm	21 (×10^9^ L^–1^)	Day 8	60 days	2 months	Oral PDN, 2 months
Baselga et al.^1^	Male	NA	11 × 13 cm	Leg	30 (×10^9^ L^–1^)	Day 12	30 days	NA	Oral PDN, 2 months
Braun et al.^3^	NA	NA	NA	NA	102 −108 g/L	Day 2-9	NA	NA	None
Andreu-Barasoain et al.^4^	Male	NA	6 cm	Arm	34 (×10^9^ L^–1^)	Day 16	1 month	1 year	Oral PDN for 1 month
Rangwala et al.^5^	Female	NA	7.5 × 9.3 cm	Arm	3 (×10^9^ L^–1^)	Day 10	2 week	1 month	A single platelet transfusion; Oral PDN for 5 days; Propranolol 1 mg/kg daily (1 month); flecainide; and a 6-week prednisolone taper.
Palma et al.	Male	NA	10 × 5 cm	Thigh	60 (×10^9^ L^–1^)	Day 4	2 weeks	2 weeks	Vitamin K and 2 infusions of fresh frozen plasma; Oral PDN for 5 days

Thrombocytopenia in RICH may correlate with the size of the tumor because it has never been reported in RICHs of less than 5 cm, ranging up to 13 cm.

A male predominance was evident (sex ratio 2.3). The lesions were located in the extremities in 63.63% of the cases, which has already been previously demonstrated that the most common CH location was on the limbs. Regarding thrombocytopenia, in all of the patients it occurred in the first days of life, resolving in the majority of cases before 2 weeks. Most of them (72.72%) were treated with oral prednisone in the range of 2 mg kg daily with a minimum of 4 days and a maximum of 2 months. Finally, 100% of the lesions regressed spontaneously within the first year of life.

In conclusion, this association may be underreported and underdiagnosed since platelet count and coagulation studies are not routinely ordered in the evaluation of RICH.

## Financial support

None declared.

## Authors’ contributions

APR, TG, CCR and YG contributed to the preparation of manuscript and critically modified. APR and TGC contributed in the preparation of figures. All authors contributed to the article and approved the submitted version.

## Conflict of interest

None declared.
